# Monitoring Sleep Changes via a Smartphone App in Bipolar Disorder: Practical Issues and Validation of a Potential Diagnostic Tool

**DOI:** 10.3389/fpsyt.2021.641241

**Published:** 2021-03-24

**Authors:** Frederike T. Fellendorf, Carlo Hamm, Nina Dalkner, Martina Platzer, Matteo C. Sattler, Susanne A. Bengesser, Melanie Lenger, Rene Pilz, Armin Birner, Robert Queissner, Adelina Tmava-Berisha, Michaela Ratzenhofer, Alexander Maget, Mireille van Poppel, Eva Z. Reininghaus

**Affiliations:** ^1^Department of Psychiatry & Psychotherapeutic Medicine, Medical University Graz, Graz, Austria; ^2^Institute of Human Movement Science, Sport and Health, University of Graz, Graz, Austria

**Keywords:** Mobile-health, bipolar disorder, smartphone app, symptom monitoring, sleep, early warning sign

## Abstract

**Background:** Sleep disturbances are common early warning signs of an episode of bipolar disorder, and early recognition can favorably impact the illness course. Symptom monitoring via a smartphone app is an inexpensive and feasible method to detect an early indication of changes such as sleep. The study aims were (1) to assess the acceptance of apps and (2) to validate sleeping times measured by the smartphone app *UP!*.

**Methods:** *UP!* was used by 22 individuals with bipolar disorder and 23 controls. Participants recorded their time of falling asleep and waking-up using *UP!* for 3 weeks. Results were compared to a validated accelerometer and the Pittsburgh Sleep Quality Index. Additionally, participants were interviewed regarding early warning signs and their feedback for apps as monitoring tools in bipolar disorder (NCT03275714).

**Results:** With *UP!*, our study did not find strong reservations concerning data protection or continual smartphone usage. Correlation analysis demonstrates *UP!* to be a valid tool for measuring falling asleep and waking-up times.

**Discussion:** Individuals with bipolar disorder assessed the measurement of sleep disturbances as an early warning sign with a smartphone as positive. The detection of early signs could change an individual's behavior and strengthen self-management. The study showed that *UP!* can be used to measure changes in sleep durations accurately. Further investigation of smartphone apps' impact to measure other early signs could significantly contribute to clinical treatment and research in the future through objective, continuous, and individual data collection.

## Introduction

Bipolar disorder (BD), a severe and lifelong mental disorder, is often misdiagnosed due to failure to identify characteristic symptoms, which leads in many cases to delayed adequate treatment strategies (1, 2). A higher number of depressive or manic episodes is associated with a lower level of functioning (3), unhealthy lifestyle, impaired cognitive function, and reduced ability to work, leading to an overall worsened course of the illness (4). Studies show early treatment with a combination of psychopharmaceuticals and psychotherapy can favorably affect the course of BD (5) and that early access to treatment is associated with shorter and milder episodes as well as longer remission (6). However, each affective episode's course and severity can vary a lot intra- and inter-individually (1, 7), and early symptoms can appear non-related to the disorder (8).

Early warning signs (EWS) are symptoms that typically occur before an affective episode (9). EWS can be observed in different modalities, including mood, thoughts, and behavior and differ inter-individually; however, individuals' unique combination of EWS is often recurring (10). Interventions taking EWS into account result in a longer time to recurrence, lower probability of hospitalization, positive impact of overall functioning (11), and are more effective than mood-stabilizing medication alone (12). One of the most common EWS is the change in sleeping patterns (13). Sleep disturbances in BD (insomnia with problems in falling asleep, sleeping interruptions, or hypersomnia) are diagnostic criteria and highly prevalent in manic and depressive episodes. Additionally, they also occur in euthymia and are associated with lower global and cognitive functioning (14), stress, dissatisfaction, and an overall decreased quality of life (15). Disturbances of the sleep-wake rhythm, in turn, can complicate the course of the episode and trigger relapse (16, 17).

Therefore, supporting patients by monitoring and noticing changes in behavior patterns early (1) is a challenge in BD's multimodal treatment (1). Usually, symptoms and behaviors are identified and recorded retrospectively through verbal explorations, questionnaires, or mood diaries. A faster and more objective, individual, and precise behavioral pattern evaluation is highly needed (18).

Mobile phones, usually available, could be an excellent opportunity for continuous recording and data collection (19). Mobile-health (m-health) is accessible for everyone, inexpensive, time-efficient, and strengthens autonomy (20, 21). One major field of m-mental health is the technology of applications (apps) for smartphones. The development of numerous m-health products provides support in screening, monitoring, and therapy in BD. Previous investigations have shown that individuals with BD have a positive attitude toward assistance tools and apps in general (22). Most of the products for BD treatment provide psychoeducational content with diverse outcomes regarding efficacy (23). Another possibility from m-health is symptom monitoring via the input of symptoms, emotional condition, or behavior.

Moreover, apps can record and process behaviors by using wireless fidelity (WIFI), accelerometer, a global positioning system (GPS), light sensors, phone and text frequency, as well as speech recognition (24–26). Objective data about exercise, physical activity, sleep, work and vacation time, and digital communication can be gathered. However, Nicholas et al. (19) found a lack of sleep monitoring with only 51% of the available monitoring apps for affective disorders measuring sleep. There is a need to validate the accuracy of measurement, the acceptance of individuals with BD, and its effectiveness as up-to-date scientific data is rare.

The aim of this study was (1) to assess the acceptance of a smartphone application (app) for individuals with BD and healthy controls (HC) and (2) to validate sleeping times within *UP!* using both a validated accelerometer and the Pittsburgh Sleep Quality Index.

## Methods

### Setting and Participants

This trial included 22 individuals with BD and 23 HC. The Department of Psychiatry and Psychotherapeutic Medicine of the Medical University of Graz conducted recruitment. Individuals with BD were either inpatients or outpatients of the dedicated outpatient center for BD. According to the Diagnostic and Statistical Manual of Mental Disorders (DSM)-IV, the diagnosis of BD was made with a structured clinical interview [SCID; (27)]. HC were recruited from the general population via written invitations and word of mouth (circle of acquaintances, medical students, clinical personnel staff). HC were screened for psychiatric diseases with a short screening questionnaire based on the SCID. Participants had to be of legal age, own an Android smartphone, and have given prior written informed consent. The trial was approved by the ethics committee of the Medical University Graz, Austria (EK-number: 29–290 ex 16/17) in compliance with the current revision of the Declaration of Helsinki, ICH guideline for GCP, and current regulations. The trial is registered at ClinicalTrials.gov as NCT03275714.

### Procedure

**Figure 1:**
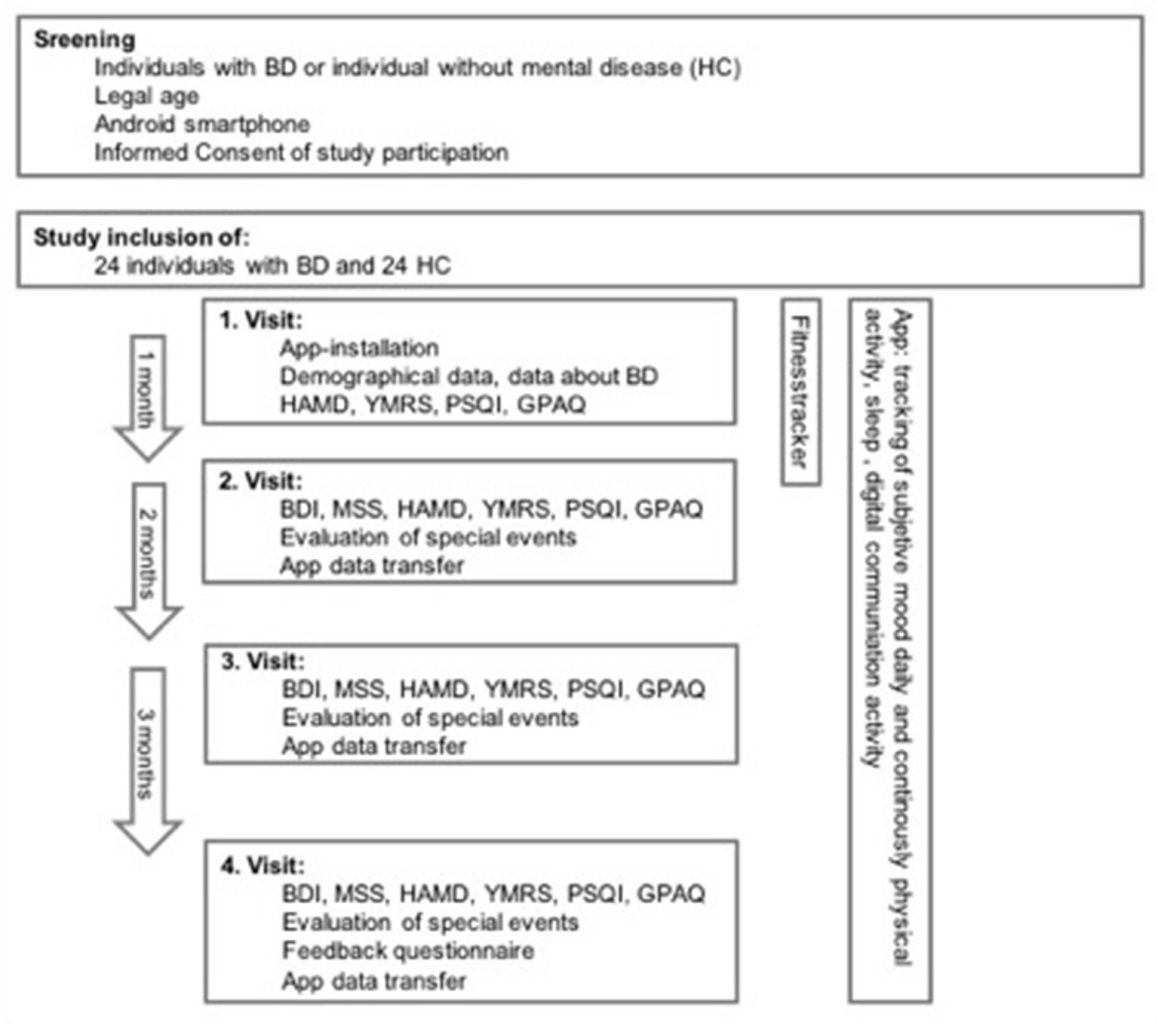
Procedure of the study.

Participants used the app *UP!* for 6 months after instructed to use their smartphone as usual. After 1 month, participants attended a scheduled visit to collect clinical data through an interview and standardized questionnaires (see Figure 1). For the first month, participants additionally wore a validated accelerometer (Axivity) on their wrist, which collected data 24 h a day for 3 weeks (due to battery and storage capacity). The study used the first month's collected data to compare the app *UP!* and the Axivity accelerometer. The first month's data collection resulted in 322 nights (of 24 participants) where both app and Axivity accelerometer measured the same nights. Demographic parameters and information about illness duration, number of episodes, and regular smartphone use were also collected. Additionally, mood symptoms from the last 2 weeks were evaluated with self-rating inventories *Becks Depression Scale* (28), *Manie-Selbstbeurteilungs-Skala* (29), and external rating scales *Hamilton Depression Scale* (30), *Young Mania Rating Scale* (31). Subjective sleep patterns were assessed by *Pittsburgh Sleep Quality Index* [*PSQI*, (32)]. Furthermore, the study participants completed a self-constructed questionnaire about observed EWS and feedback about the app.

### Material

The Android Smartphone software solution *UP!* was developed by *meemo-tec OG* with the medical consult of the dedicated outpatient center for BD, Graz, Austria. Automatic data collection includes sleep, physical activity, and social profiles. The sleeping behavior of the user is recorded using the phone's accelerometer and light sensors. As a result, real sleeping events are distinguishable from simple inactivity. The app determines the time of falling asleep and waking up and time slept during the day. Moreover, users were asked to rate their mood with seven smiley-emoticon options once daily in the evening. Data were extracted and converted by meemo-tec software. Timestamps were used to record minutes of sleep disruption activity via timestamps of falling asleep and waking up for single nights.

The accelerometer *Axivity3* [*AX3, Axivity, Newcastle upon Tyne, UK*, (33)] is a triaxial accelerometer worn on the wrist of the non-dominant hand. This waterproof device consists of a flash-based memory, a real-time quartz watch that was not visible for users, and a temperature sensor. The tracker was validated in assessing physical activity behaviors as well as sleep periods in adults (34). In our study, the acceleration was recorded at 50 Hz. The devices were programmed to start measuring 1 week after the first visit due to the maximum storage capacity of 3 weeks using 50 Hz (based on data from pilot tests). Raw data were extracted using GENEActiv PC software version 3.2. Signal processing was performed in R (version 3.5.1; http://cran.r-project.org) using package GGIR (version 1.6–7). A description of the analysis and applied algorithms can be found elsewhere (35). Briefly, the vector magnitude (expressed in mg) using the Euclidean norm minus 1 g (ENMO) was calculated for each 5-s epoch. The non-wear periods were defined by windows of 60 min. The sleeping duration was estimated using the Change in Z-Angle algorithm (34).

The *PSQI* by Buysse et al. (32) is a self-rated questionnaire, which assesses sleep quality and disturbances over a 1-month time interval. The questionnaire consists of 19 items, which generate seven components: subjective sleep quality, sleep latency, sleep duration, habitual sleep efficiency, sleep disturbances, use of sleep medication, and daytime sleepiness. Each component scores from 0 (“no difficulty”) to 3 (“severe difficulty”). A total PSQI score (range 0–21) of more than 5 distinguishes good and poor sleepers, whereas higher scores indicate worse sleep quality.

The *self-constructed questionnaire* in the German language contained, among others, questions about EWS as “Do you recognize when a depressive episode begins?,” “Do you notice the same signs again and again at the beginning of a depressive/(hypo)manic episode?,” “If yes, which of the following occur in more than half of the episodes? (Multiple answers possible).” Furthermore, questions about app use were: “What apps do you use on your smartphone?,” “Do you think a technical support in the treatment of BD is meaningful?,” “How often do you check your smartphone?,” and “Does your response time in texting change in depressive/(hypo)manic episodes?.” Moreover, questions about study participation included: “Did you find the app annoying?,” “Have you been concerned about the protection of your personal data?,” “Can the app be integrated into everyday life?,” “Does the measurement of sleep/movement/working time via smartphone make sense in BD treatment?,” “Do you desire graphical feedback of behavior measured by the app,?” and “Would you use this app as a support tool in BD treatment?-reasons for yes/no.”

### Statistical Analyses

All analyses were performed with the IBM Statistical Package for Social Sciences (SPSS), version 26.0. Unpaired *t*-tests (metric data), Mann–Whitney *U*-tests (ordinal data; metric data not normally distributed), and chi-square tests were conducted to test for differences between the BD and HC group in descriptive variables and acceptance. Wilcoxon tests investigated the difference of mood inventory scores between the start and the end of the trial. Due to technical issues in the clinical trial setting, there were only sleeping times of 24 participants (12 with BD, 12 HC) available within the app. Pearson correlation coefficients were calculated to compare and validate falling asleep and waking-up times for the three measurement methods: the app with accelerometer and app with PSQI. For the correlations between the app and the accelerometer, all nights were included when the app and accelerometer data were coincidently available (*n* = 322). As the PSQI only measures a mean of the last 4 weeks, for the analyses between app and PSQI, means of the app data were built first and then correlated (*n* = 24). Error probabilities of *p* < 0.05 were accepted. Bonferroni corrections were adjusted in correlation analyses for the four sleeping time tests (0.05/4 tests = 0.0125).

## Results

Sociodemographic and illness-specific data regarding the number of episodes and current treatment of the participants can be observed in Table 1. Individuals with BD differed significantly in age to HC, while the proportion of females and males did not differ between the groups. There were significant differences in HAMD and YMRS scores between individuals with BD at the beginning of the study and after 1 month; still, all values did not correspond to clinically relevant depressive nor manic symptomatology (Table 1). In the patient group, the HAMD improved significantly from the start of the trial to the end after 6 months (*Z* = −2.139; *p* = 0.032). There was no significant difference in YMRS.

**Table 1:** Sociodemographic and illness specific data of individuals with BD and HC.

	**Individuals with BD (*n* = 22)**	**HC (*n* = 23)**	**Value**	***p***
Sex	54.5% male	43.5% male	*X*^2^(1) = 0.018	0.894
	45.5% female	56.5% female		
Age	43.36 (±10.89)	35.00 (±11.39)	*U* = 148.50	0.018^*^
Diagnosis of BD (age)	32.32 (±13.41)	–		
Number of depressive episodes	6.82 (±4.64)	–		
Number of (hypo)manic episodes	4.23 (±3.52)	–		
Current treatment		–		
- Psychopharmaceuticals	86.4%			
- Psychotherapy	54.5%			
- Psychoeducation	18.2%			
- Self-help group	27.3%			
- Other (e.g., acupuncture, painting, exercise)	27.3%			
Mood inventories:				
- HAMD t1	6.18 (±5.89)	0.26 (±0.45)	*U* = 49.50	<0.001^**^
- YMRS t1	3.23 (±4.89)	0.04 (±0.21)	*U* = 109.50	<0.001^**^
- HAMD t2	4.82 (±5.78)	0.96 (±1.11)	*U* = 104.50	0.001^**^
- YMRS t2	2.27 (±3.09)	0.00 (±0)	*U* = 126.50	<0.001^**^
- HAMD t3	3.50 (±3.85)	1.60 (±2.77)	*U* = 79.50	0.044^*^
- YMRS t4	1.06 (±2.10)	0.00 (±0)	*U* = 90.00	0.108

BD, bipolar disorder; HC, healthy controls; HAMD, Hamilton Depression Scale; YMRS, Young Manie Rating Scale; t1, start; t2, 1 month after start; t2, 6 months after start;

* p < 0.05;

** *p < 0.01. Significant results are presented in bold*.

Table 2 demonstrates how often participants checked their smartphone for messages and whether they used apps on it. Participants with BD and HC did not differ in most of the apps used on the smartphone. Only communication apps were used more by HC. Individuals with BD stated that the response latency to messages lengthened considerably during the onset of depressive episodes. Two-thirds of individuals with BD stated that the response latency decreased significantly during the onset of (hypo)manic episodes.

**Table 2:** Smartphone usage of individuals with BD and HC.

	**Individuals with BD (*n* = 22)**	**HC (*n* = 23)**	**Value**	***p***
App usage on smartphone	100%	100%		
- Facebook	54.5%	43.5%	*X*^2^(1) = 0.55	0.458
- Whatsapp	95.5%	91.3%	*X*^2^(1) = 0.31	0.577
- Other communication (e.g., Skype, Viber)	18.2%	52.2%	*X*^2^(1) = 5.67	0.017^*^
- Physical activity (e.g., Runtastic)	13.6%	26.1%	*X*^2^(1) = 1.09	0.297
- Sleep	0%	8.7%	*X*^2^(1) = 2.00	0.157
- Others	36.4%	39.1%	*X*^2^(1) = 16.99	0.386
Smartphone check during week			*X*^2^(4) = 4.46	0.348
- Only when ringing	18.2%	4.3%		
−1–2 times a day	0%	4.3%		
−3–4 times a day	31.8%	26.1%		
- Almost every hour	36.4%	34.8%		
- Several times an hour	13.6%	30.4%		
Smartphone check during weekend			*X*^2^(2) = 4.10	0.353
Only when ringing	18.2%	4.3%		
−1–2 times a day	0%	8.7%		
−3–4 times a day	36.4%	30.4%		
- Almost every hour	31.8%	34.8%		
- Several times an hour	13.6%	21.7%		
Almost instant reply to messages	50%			
**Change in response time in depressed mood**				
- No	16.7%			
- Yes, extended	75.0%			
- Yes, shortened	8.3%			
**Change in response time in euphoric mood**				
- No	33.3%			
- Yes, extended	8.3%			
- Yes, shortened	58.3%			

BD, bipolar disorder; HC, healthy controls;

* *p < 0.05. Significant results are presented in bold*.

Table 3 depicts the times of falling asleep and waking up measured using the PSQI, the app *UP!*, and the Axivity accelerometer. There were no differences between individuals with BD and HC in falling asleep and waking-up times during testing. Additionally, no difference in sleeping times variblities were found between the groups [app falling asleep: *M*_*Pa*t_ = 1:19, *M*_*HC*_ = 1:16, *T*_(25)_ = 0.230, *p* = 0.820; app waking-up: *M*_*Pa*t_ = 1:11, *M*_*HC*_ = 1:13, *T*_(25)_ = −0.255, *p* = 0.801; accelerometer falling asleep: *M*_*Pa*t_ = 1:13, *M*_*HC*_ = 1:13, *T*_(39)_ = 0.022, *p* = 0.983; accelerometer waking-up: *M*_*Pa*t_ = 1:08, *M*_*HC*_ = 1:04, *T*_(39)_ = 0.431, *p* = 0.669].

**Table 3:** Sleeping times measured with PSQI, *UP!* app, and Axivity accelerometer in individuals with BD and HC.

	**Individuals with BD (M ± SD)**	**HC (M ± SD)**	**Value**	***p***
**PSQI (*****n***_**BD**_ **=** **22**, ***n***_**HC**_ **=** **23)**				
- Time falling asleep	22:45 (±1:45)	22:41 (±0:41)	*U* = 243.50	0.827
- Time waking up	6:52 (±1:12)	6:46 (±0:43)	*U* = 247.50	0.900
- Sleeping duration (hours)	7.18 (±1.78)	6.92 (±1.21)	*U* = 204.00	0.260
- Duration falling asleep (min)	24.75 (±26.69)	18.57(±11.97)	*U* = 247.50	0.900
- Subjective sleep quality	0.95 (±0.72)	0.91(± 0.60)	*U* = 246.00	0.858
- Sleep disturbances	1.00 (±0.54)	1.04(±0.37)	*T*(43) = −0.32	0.751
- Sum score	6.95 (±4.38)	5.26(±2.83)	*U* = 195.00	0.185
**App (*****n***_**BD**_ **=** **12**, ***n***_**HC**_ **=** **15)**				
- Time falling asleep	23:03 (±1:00)	23:06 (± 0:51)	*U* = 807.50	0.903
- Time waking up	7:31 (±0:51)	7:09 (0:46)	*T*(25) = 1.16	0.257
**Tracker (*****n***_**BD**_ **=** **21**, ***n***_**HC**_ **=** **20)**				
- Time falling asleep	23:35 (±1:25)	23:33 (±1:11)	*T*(39) = 0.08	0.934
- Time waking up	7:15 (±0:51)	7:20 (±0:59)	*T*(39) = −0.28	0.778

*BD, bipolar disorder; HC, healthy controls; PSQI, Pittsburgh Sleep Quality Index; M, mean (of sleeping times); SD, standard deviation*.

For correlations between the app and accelerometer, all coincident data nights were analyzed using the whole group of individuals included in the study. As PSQI sleeping times are parameters for the last 4 weeks, the mean values of the app's data were used. The significant correlation between the app's and the accelerometer falling asleep times correspond to a high validity (*n*_nights_ = 322; *r* = 0.77, *p* < 0.001). There was a high correlation between the app and the PSQI in falling asleep times (*n*_participants_ = 24, *r* = 0.64, *p* = 0.001). There were also significant high correlations for the waking-up time of the app and the accelerometer (*n*_nights_ = 322; *r* = 0.59, *p* < 0.001), as well as between the app and the PSQI (*n*_participants_ = 24, *r* = 0.53, *p* = 0.007). All correlations survived Bonferroni corrections (0.05/4 tests = 0.0125).

Individuals with BD positively evaluated the app *UP!* for measuring sleep, physical activity, and working duration (see Table 4). Moreover, participants reported they found a graphical feedback of their behavior measured by an app to be helpful. Most of the interviewed individuals stated that they would use an app as a support tool in BD treatment, mainly because of continual sleep observance, awareness for changes due to graphical presentation/feedback, and easy integration into everyday life. Most individuals with BD did not perceive the app *UP!* as “annoying.” For three BD individuals who reported annoyance, this was due to wearing the Axivity accelerometer (which was only done for the study as the app would be used as a standalone post-study). No participant was annoyed by having the smartphone around all the time. One individual with BD reported being annoyed by other things such as “active GPS” and “high battery need.”

**Table 4:** Desire for an app in BD treatment.

	**Individuals with BD (*n* = 18)**	**HC (*n* = 15)**	**Value**	***p***
**Did you find the app annoying?**				
- No	81.8%	47.8%	*X*^2^(3) = 6.07	0.103
- Rare, <1/week	4.5%	17.4%		
- Sometimes, 1–2/week	13.6%	30.4%		
- Often, almost daily	0%	4.3%		
Have you been concerned about the protection of your personal data? (Yes)	13.6%	21.7%	*X*^2^(1) = 0.51	0.477
Can the app be integrated into everyday life? (Yes)	90.9%	100%		
Does the measurement of movement via smartphone make sense in BD treatment? (Yes)	100%	93.3%	*X*^2^(1) = 1.24	0.266
Does the measurement of sleep via smartphone make sense in BD treatment? (Yes)	100%	100%		
Does the measurement of working duration via smartphone make sense in BD treatment? (Yes)	88.9%	93.3%	*X*^2^(1) = 0.20	0.658
Desire for graphical feedback (Yes)	100%	86.7%	*X*^2^(1) = 2.56	0.110
**Would you use this app as a support tool in BD treatment? (Yes)**				
Reasons for use	83.3%	100%	*X*^2^(1) = 2.75	0.097
- Too little everyday overview with current treatment	33.3%	37.5%	*X*^2^(1) = 0.68	0.409
- Continual sleep observance	60.0%	40.%	*X*^2^(1) = 0.60	0.439
- Continual movement observance	20.0%	20.0%	*X*^2^(1) = 0.00	>0.999
- Continual mood observance	46.7%	46.7%	*X*^2^(1) = 0.00	>0.999
- Awareness for changes due to graphical presentation/feedback	73.3%	80.0%	*X*^2^(1) = 0.19	0.666
- Easily integrable into everyday life	73.3%	73.3%	*X*^2^(1) = 0.00	>0.999

*BD, bipolar disorder; HC, healthy controls*.

## Discussion

This study aimed to determine whether individuals with BD consider technical support through a smartphone app to be useful and practical in the early detection of symptoms during BD treatment. Individuals with BD assessed the monitoring of EWS with a smartphone as positive. Our investigation did not register strong reservations concerning data protection or continual smartphone usage. Moreover, correlations demonstrate that the app *UP!* is a valid tool for measuring falling asleep and waking-up times.

The earlier EWS of both depressive and (hypo)manic episodes are recognized, the better the prognosis for the course of the individual episode and thus the influence on lifelong illness (9). Psychotherapeutic and self-effective interventions used to regulate sleep behavior, physical activity, and a balance in social activities could counteract the development of disease-related symptoms at an early stage (36). Consequently, the detection of EWS should be a focus in the treatment of BD. Using smartphones, EWS, presenting in mood, symptoms, and behavior changes such as sleep disturbances, may be detected more quickly and better tailored to the individual. Access to the detection of these changes could lead to early self-recognition of EWS and the users' faster reactions and, therefore, increase self-efficacy. It seems likely that innovative products could bring advantage as an add-on or self-contained therapy. Although there was an improvement in HAMD in individuals with BD, the small values do not represent depressive symptomology. Moreover, many other factors influenced mood besides the inventions of the trial. In sum, with these results, a conclusion on the impact of the app alone on illness symptomatology cannot be drawn.

Individuals with BD stated that sleep is the most critical pattern to monitor with an app (37). We hypothesized in this study that smartphone apps could reliably measure sleep patterns and changes in these patterns equivalent to EWS. Our results showed *UP!* to be a valid option for that. The aim should be to detect EWS based on objective, continuous, and individual data. Kolla et al. (38) stated that accelerometers and apps underestimate sleep disruptions and overestimate total sleep times compared with polysomnography. However, EWS detection's target is the change of individual behavior rather than registering the accurate falling asleep and waking-up time. The recorded behavior patterns are presented to the users in different ways, depending on the product. For example, the advanced version of *UP!* offers a graphic overview of sleeping times, working hours, physical activity, smartphone usage time, and mood. Just the visualization and knowledge of behaviors, patterns, and changes can change the mindset of those affected and reinforce “healthy behavior” such as a balanced sleep-wake rhythm or sufficient movement. By increasing self-management activities, depressive symptoms can be reduced (39). Moreover, the daily use of apps can strengthen the introspective abilities. However, the efficacy is dependent on regular use of the monitoring apps, also in euthymia.

In addition, many products alert users when pathological parameters, such as no or very little sleep or a very bad mood, are measured. The system then automatically asks users about other symptoms from a list of general or individual EWS. A few first trials were conducted to develop learning systems based on machine learning techniques (40). This development aims to recognize EWS and notifying patients on a very individual basis. Presently, a realistic goal of apps in BD treatment is to improve self-management, which should ultimately prevent recurrences, relapses, severe episodes, and hospital stays.

The results support the finding of a larger trial showing that a substantial proportion of individuals with BD appreciate an app that measures EWS in particular sleep and would use it in BD treatment. In 2014, a study group in the USA also examined 320 people with a mental disorder and found that 62.5% had a smartphone, and 70.6% of those were interested in using it to monitor symptoms (41). A survey of 89 individuals with BD showed that 40% already used apps for managing illness symptoms, and 79% of the others would like to use specific tools (42). Participants reported wanting self-management tools, sleep-management, EWS, and triggers, emphasizing easy usage, scientific quality, and data privacy. However, the same study group found in a review that a large part of the available apps for BD do not meet these needs (43). A recent survey of 47 individuals with BD found that more were adherent to a smartphone app than a Fitbit fitness tracker (37). Similarly, participants in this study rated the wearing of the Axivity accelerometer as more annoying than using an app. Moreover, Van Til et al. (37) surveyed that individuals with BD would like to have monthly personal talk with their clinician in case of using an app. However, presumably subgroups of individuals with BD prefer apps and/or accelerometers. Factors such as age, sex, mobile phone use, and psychopharmacological and psychotherapic treatment should be considered in more extensive trials.

In general, no direct negative impact is to be expected from using an app. Apps should always comply with data protection regulations. Participants in this study did not have any concerns about the misuse of their personal data. Most of the available apps store the data locally on the smartphone. Directly sending data to clinicians would result in data protection problems and lead to potential liability issues. There is also evidence of adverse effects of EWS interventions. While individuals with BD might become over-confident with self-management strategies and stop their needed medication (12), recognizing EWS of depression might lead to rumination and worsening depressive symptomatology (44).

Monitoring via an app could bring additional advantages to treat BD. First, links for psychoeducational tools could be implemented within apps. Bauer et al. (45) showed that individuals with BD often use the Internet to inform themselves about their disease. It seems very likely that individuals with BD would appreciate and use direct links via smartphones. Second, it would be an asset for clinicians if individuals with BD could provide a structured data format of their symptoms at their physicians' appointments. In the past, physicians, therapists, and researchers have had to rely on subjective, retrospective information. A recent survey stated that health care professionals' attitudes toward using apps in clinical practice are quite positive. However, the knowledge of technology and products and consequently the use in their daily clinical practice differed a lot (39). Third, assessing valid, objective data on behavioral patterns and monitoring them over a longer time is necessary for clinical research (25, 46) and could be used to further understand and treat BD.

Even though it is unlikely and not desirable that m-mental health products will replace medical and therapeutic treatments, treatment teams, and scientists have to evaluate the extent to which m-mental health could be a useful support tool in BD. One major challenge in the near future is to validate products' effectiveness on symptom reduction, well-being, and hospitalization rate. There are already many m-health products tailored for BD available. So far, a few study protocols for clinical validation and evaluation studies have been published, but final results and efficacy assessments are still insufficient (47–50). Therefore, further aims of studies may be to test whether apps are valid and whether recorded changes in behavior are associated with mood changes and other symptoms of the disease.

### Limitations

There are several limitations of this study. Noticing EWS and acceptance of the app were self-conducted variables with no reference value. Furthermore, the sample size is relatively small. Due to technical problems in the clinical setting, the app did not measure all participants' sleeping data. Additionally, it was not possible to compare sleep interruptions as the *UP!* and Axivity accelerometer did not comparably analyze this data. However, sleep duration seems to be more relevant as EWS than interruptions. As the study duration was only 6 months, the frequency of affective episodes is relatively small. Further studies to investigate the impact of using apps on sleep, behavior, and illness symptomatology are therefore necessary.

## Conclusion

Individuals with BD as well as clinicians would benefit from additional options in BD treatment. Individuals with BD assessed the measurement of sleep as an early warning sign with a smartphone as positive. Detecting early signs is essential for improving the course of the disease and could change individuals' behavior and strengthen self-management. Thus, in the future, validated smartphone apps can significantly contribute to clinical treatment and research through objective, continuous, and individual data collection. The study showed that *UP!* can be used to measure changes in sleep durations accurately. The validity for measuring other early signs needs to be investigated further. Even if it is unlikely that m-health products will completely replace medical and therapeutic treatments, they can represent an additional treatment strategy. The development, validation, and evaluation of specific products' effectiveness will be necessary for the future.

## Data Availability Statement

The raw data supporting the conclusions of this article will be made available by the authors, without undue reservation.

## Ethics Statement

The studies involving human participants were reviewed and approved by Medical University Graz. The patients/participants provided their written informed consent to participate in this study.

## Author Contributions

FF has designed the study, written the first and last draft, and was responsible for the study conception, coordination, and publication of data. CH and RP were involved in study coordination and data collection. ND and MPl were involved in the conception of the study and writing. In addition, MPl supervised and guided us through the whole process of publication. MS and MPo were involved in study conception and technical support of data of the accelerometer. SB, ML, AB, RQ, AT-B, MR, and AM were responsible for proofreading and revising the manuscript. ER supervised the whole study procedure and revised for important intellectual content. All authors contributed to the article and approved the submitted version.

## Conflict of Interest

The authors declare that the research was conducted in the absence of any commercial or financial relationships that could be construed as a potential conflict of interest.
